# Digenic mutations on *SCAP* and *AGXT2* predispose to premature myocardial infarction

**DOI:** 10.18632/oncotarget.22045

**Published:** 2017-10-24

**Authors:** Yuanfeng Gao, Chongyou Lee, Junxian Song, Sufang Li, Yuxia Cui, Yongzhen Liu, Jie Wang, Fengmin Lu, Hong Chen

**Affiliations:** ^1^ Department of Cardiology, Peking University People's Hospital, Xicheng District, Beijing, China; ^2^ Beijing Key Laboratory of Early Prediction and Intervention of Acute Myocardial Infarction, Peking University People’ s Hospital, Beijing, China; ^3^ Center for Cardiovascular Translational Research, Peking University People’s Hospital, Beijing, China; ^4^ Department of Microbiology and Infectious Disease Center, Peking University, Beijing, China

**Keywords:** premature myocardial infarction, genetics, exome-sequencing, CRISPR-Cas9, digenic mutation

## Abstract

Genetic factors play a vital role in the pathogenesis of premature myocardial infarction (PMI). However, current studies explained only small amounts of genetic risk in MI. In this study, we started from a PMI pedigree with three MI patients occurred at the age of 43, 45 and 53 respectively. Sanger sequencing revealed 6 LDLR mutation carriers in the family, but only one was diagnosed with PMI, indicating that the LDLR mutation may not be the reason for PMI. Upon exome-sequencing and bioinformatics analysis, two variants in *SCAP* and *AGXT2* were identified as potential causative mutation for PMI. Further observation revealed that only patients that meet the diagnosis of PMI harbored two variants meantime, while other MI patients or members with no MI carried no more than one of the variants. Screening of the two genes in an independent PMI population identified another variant on *SCAP* (c.1403 T>C, p.Val468Ala), which was absent in 28, 000 east-Asian population. Further, the two variants on *SCAP* and *AGXT2* were introduced into H293T and EA. hy926 cell lines respectively utilizing CRISPR-Cas9. Functional study revealed that the *SCAP* mutation impaired SCAP-SREBP feedback mechanism which may lead to a “constitutive activation” effect of cholesterol synthesis related genes, while the *AGXT2* mutation reduced its aminotransferase activity leading to a down-regulation of NO production by ADMA accumulation. This study indicates that *SCAP* and *AGXT2* are potential causative genes for PMI. Digenic mutation carriers may manifest a more severe phenotype, namely premature MI.

## INTRODUCTION

Myocardial Infarction (MI) is the most severe type of Coronary Artery Disease (CAD), ranking the leading cause of death worldwide [1, 2]. As a complex disease, genetic factor, the extent of which is defined by heritability, exerts a major influence on the cause of MI. Especially the premature MI, its heritability (63%) is significantly higher than normal MI as seen from the results of twin study on MI [3, 4]. During the past 10 years, Genome-wide Association Study (GWAS), mainly by examining the contribution of common variants of single nucleotide polymorphisms (SNPs) (minor-allele frequency >5%) for association with diseases, has been the most popular measure in the endeavor for genetic study of CAD or MI. During the past 10 years, 60 loci have been reported to have roboust links to risk of CAD by this genetic association [5]. Approximately one third of the Loci that were found in GWAS have been attributed to relate to known and putative risk factors of CAD, like hypertension and hypercholesterolemia, and most of the molecular mechanisms of the Loci in pathogenesis of CAD have not been elucidated [6]. Besides, despite the involvement of tens of thousands cases and controls, a large fraction of the genetic risk are still yet to be found out [7]. With the arrival and ever-evolving of Next generation sequencing (NGS), genetic study has been enormously facilitated. Instead of using a SNP microarray for genotyping in GWAS, NGS could “read” the sequence of the individual genome directly, including the rare variants that may be disease generating. For example, a NGS-based study on CAD was carried out in 2015, they scanned each gene of ∼5000 cases with early-onset CAD to controls without CAD in order to find genes of significant associations with CAD, the result showed that 2% of studied early-onset CAD patients harbored at least a rare variant on *LDLR*, which is a long recognized gene relating to hypercholesterolemia and CAD [8]. This finding justified the effectiveness of NGS in finding rare variants that related to diseases. However, because of the low minor-allele frequency of rare variants, it would need thousands of cases and controls to achieve statistical power.

Nevertheless, due to the homogeneity of genetic background in a family, the disease-causative variants may be of the same origin on a given family. When combined NGS with family-based study, it could help screen out the potential causative mutations in the affected family members directly by sequencing fewer individuals. So the present study aims to investigate the causative genes of MI in a pedigree with multiple members with PMI by whole-exome sequencing. Population and functional studies were then invested to further support the MI-causative role of the potential pathogenic genes found in the present study.

## RESULTS

### Clinical characteristics of the index patient and her family members

The index patient (III.6), a 42-year-old woman, was diagnosed to have an acute myocardial infarction at the age of 39. The diagnoses of coronary heart disease, hypercholesterolemia as well as hypertension was made when she was 35. Family history inquiring reveals that his elder brother (III.3) and uncle (II.3) were also diagnosed to have MI at the age of 42 and 53 respectively. Further evaluation shows that several members of the family could have been diagnosed as hypercholesterolemia (Table 1), especially in the paternal line. So we carried out sanger sequencing to investigate whether family hypercholesterolemia (FH) is the cause of the multiple MI victims in the pedigree.

**Table 1:** General information of all the family members. (n=16)

ID	Sex	Age	Lipid profile (mmol/l)				MI history (Age of onset)	Hypertension
			TC	LDL-C	HDL-C	TG		
II.1	Male	73	8.22	5.75	1.33	2.7	No	-
II.2	Female	68	7.77	4.76	1.34	4.22	No	+
II.3	Male	67	6.99	4.12	1.49	2.12	53	-
II.4	Female	70	5.59	3.58	1.38	1.12	No	-
II.5	Male	68	7.4	5.54	1.3	1.61	No	-
II-6	Female	58	5.34	2.85	1.28	5.02	No	-
II-7	Male	65	6.66	4.86	0.91	1.72	No	-
III.1	Male	50	6.45	4.08	1.46	2.62	No	+
III.3	Male	48	5.71	3.69	0.81	3.98	42	+
III.4	Female	41	5.59	3.58	1.38	1.12	No	-
III.5	Male	43	4.3	3.05	0.7	2.59	No	-
III.6	Female	43	8.28	5.04	1.02	5.04	39	+
III.7	Female	47	6.2	4.69	1.2	0.8	No	-
III.8	Female	41	5.7	3.87	1.4	1.31	No	-
IV.1	Male	28	4.91	2.4	1.73	0.62	No	-
IV.2	Male	20	5.45	3.9	1.11	1.56	No	-

### Genetic screening of *LDLR* found a potential disease-causive mutation

*LDLR* was reported to be the most frequently related gene to hypercholesterolemia as well as early-onset CAD [8], so we first carried out sanger sequencing of *LDLR* on the index patient (III.6). It turned out that she harbored a potential FH causative mutation of c.1432G>A (p.Gly478Arg) on Exon10 of *LDLR*. (Figure 2) The very mutation has been found to associate with FH based on the Leiden Open Variation Database of FH (http://www.ucl.ac.uk/ldlr/Current/). Further genotyping of the family members detected another 5 *LDLR* mutation carriers. However, the *LDLR* mutation does not co-segregate with the MI phenotype. Especially that the other PMI patient (III.3) was not found to have a FH causing mutation when other three *LDLR* mutation carriers had no coronary heart disease symptoms or radiography changes. This indicated that there may be other underlying deleterious mutations that may have caused the PMI phenotype in this family. So we further employed whole-exome sequencing on the two PMI patients to find new MI causing genes.

**Figure 1:**
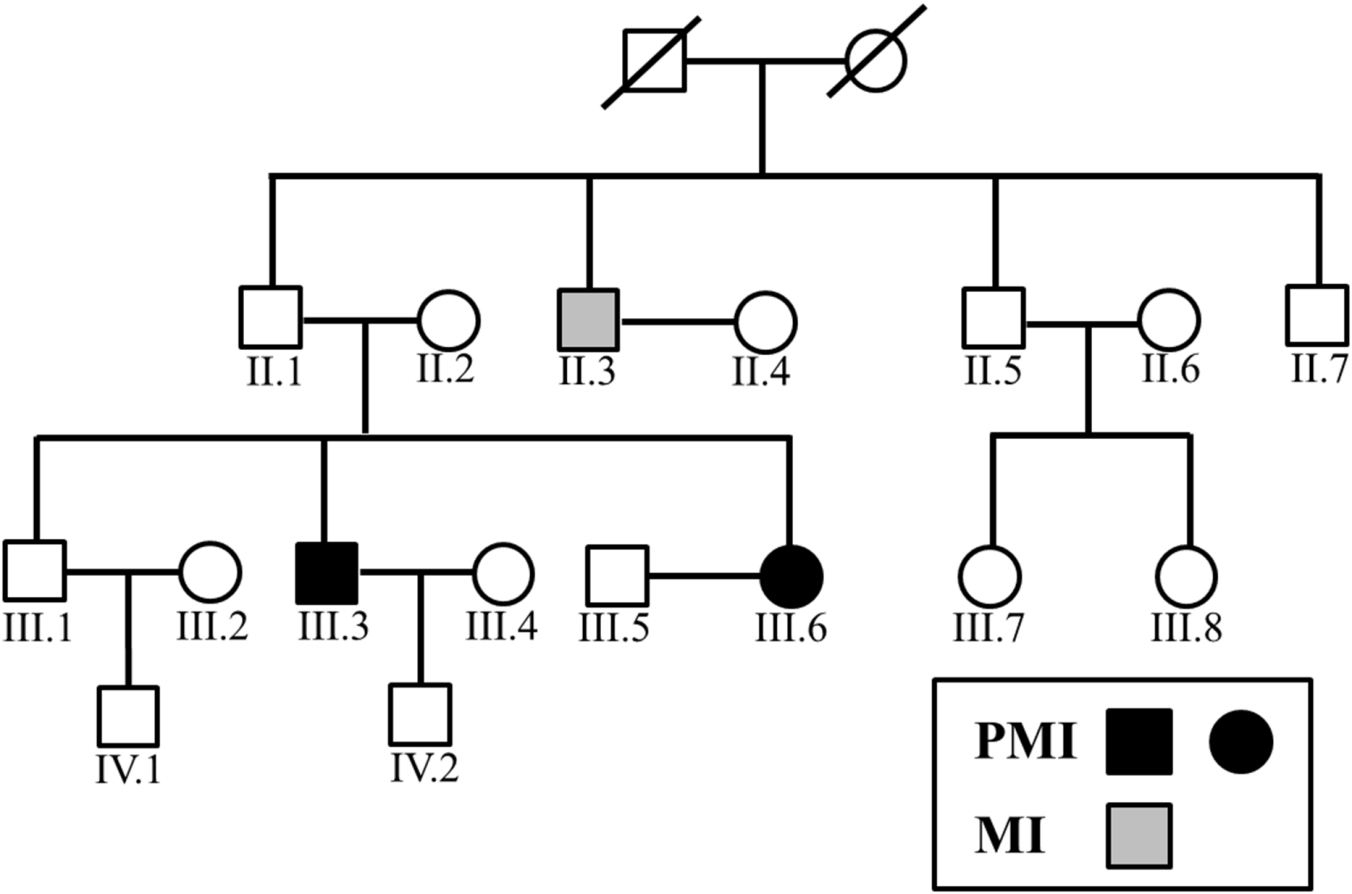
Family tree of the PMI pedigree

**Figure 2:**
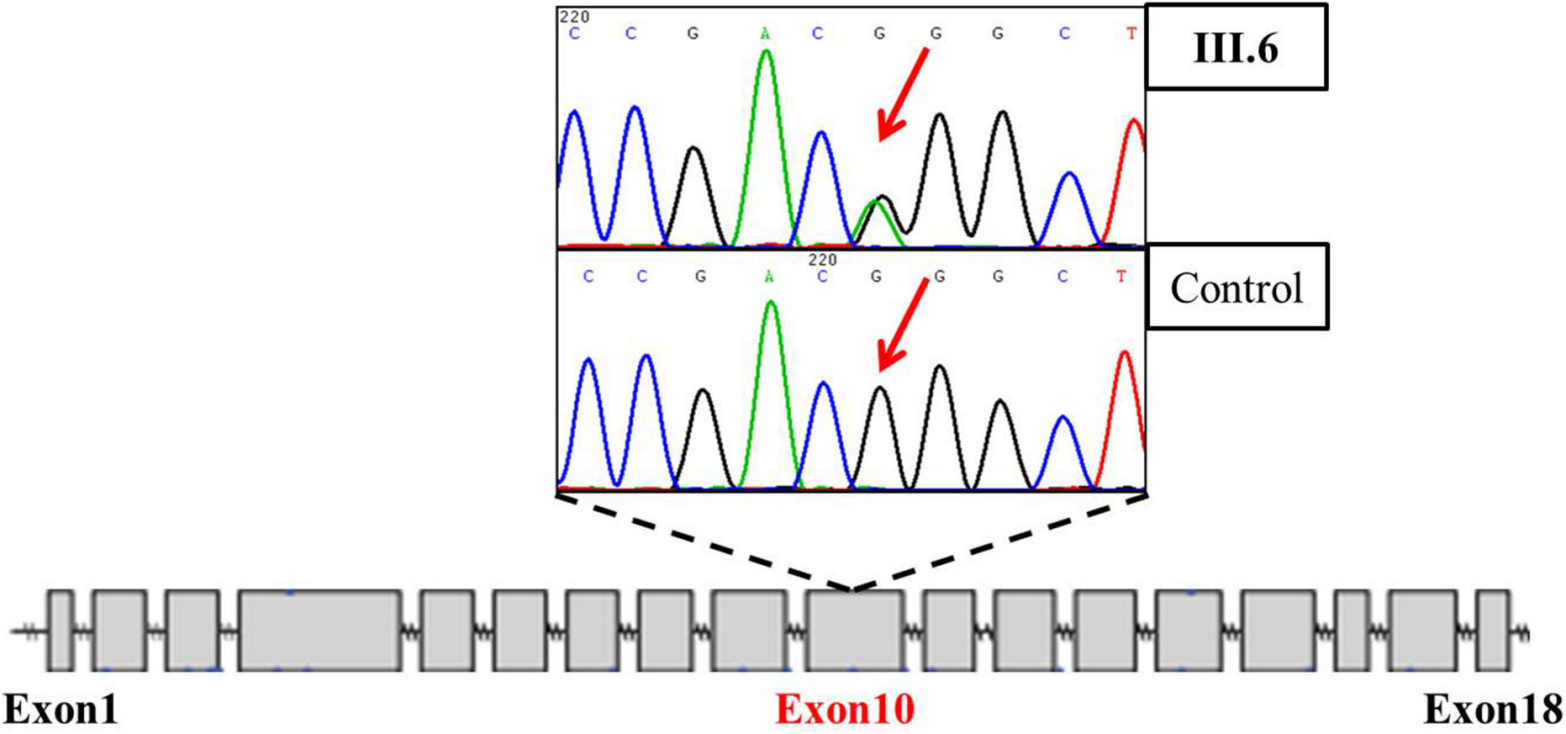
Truncated sequencing chtomatogram of *LDLR* of III. 6 (index patient diagnosed with PMI)

### Whole-exome sequencing reveals two new potential PMI causing mutations

**Figure 3:**
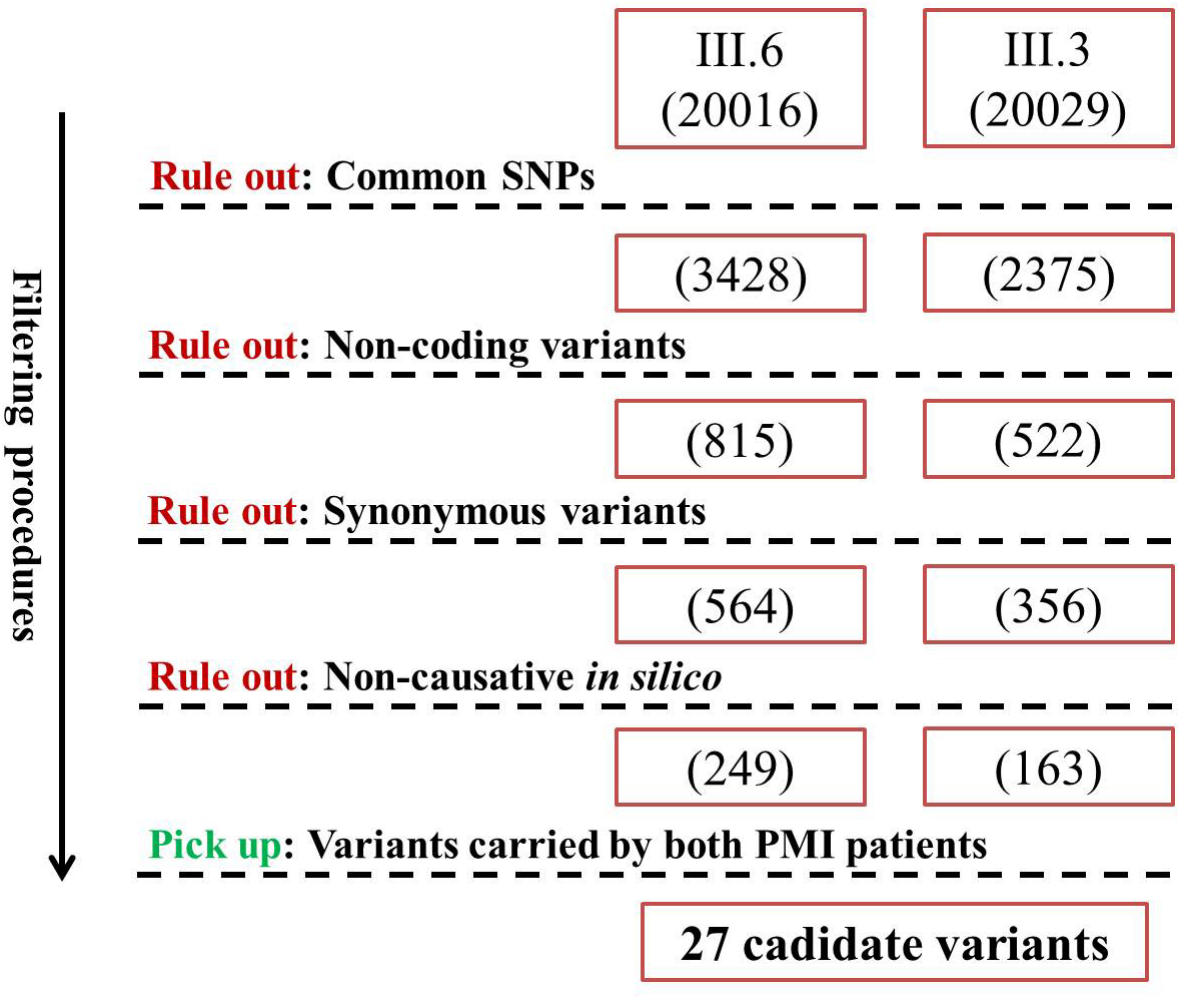
Filtering strategy for candidate variants of PMI Numbers in brackets denote the amount of remaining variants after each filtering procedure.

The two PMI patients were detected to have 20016 and 20029 variants by Whole-exome sequencing. We then filtered the variants to rule out the variants that were not likely to alter gene function, leaving 27 candidate variants (Figure 3). Then we utilized two strategies to further select the potential causing mutations of PMI among these variants. Firstly, we utilized Phenolyzer [9] (http://phenolyzer.wglab.org/) to filtrate the candidate genes by imputing “premature/early-onset myocardial infarction” and symbols of the 27 genes. Eight genes (including *AGXT2*, *TK2*, *SRP72*, *SLC4A9*, *SCAP*, *PPP2R5B*, *COL7A1*, *NEFL*) were reported to be potential PMI related genes Figure 6. Meanwhile, we searched the potential association of the 27 candidate genes in pubmed using search strategy (atherosclerosis or “myocardial infarction” or “coronary heart disease”) and symbols of the 27 genes. Four genes (including *AGXT2*, ZFHX3, *SCAP*, *TCF4*) were found to be related to the PMI related. It turned out to be that only *SCAP*-c.3035C>T (p.Ala1012Val) and *AGXT2*-c.1103C>T (p.Ala338Val) were predicted to be causive by both strategies. In addition, after screening the two variants in other family members, only subjects carrying both variants were PMI victims, while individuals with no more than one variant were have no MI or PMI. We then subjected the two potential mutations to population and functional verification.

**Figure 4:**
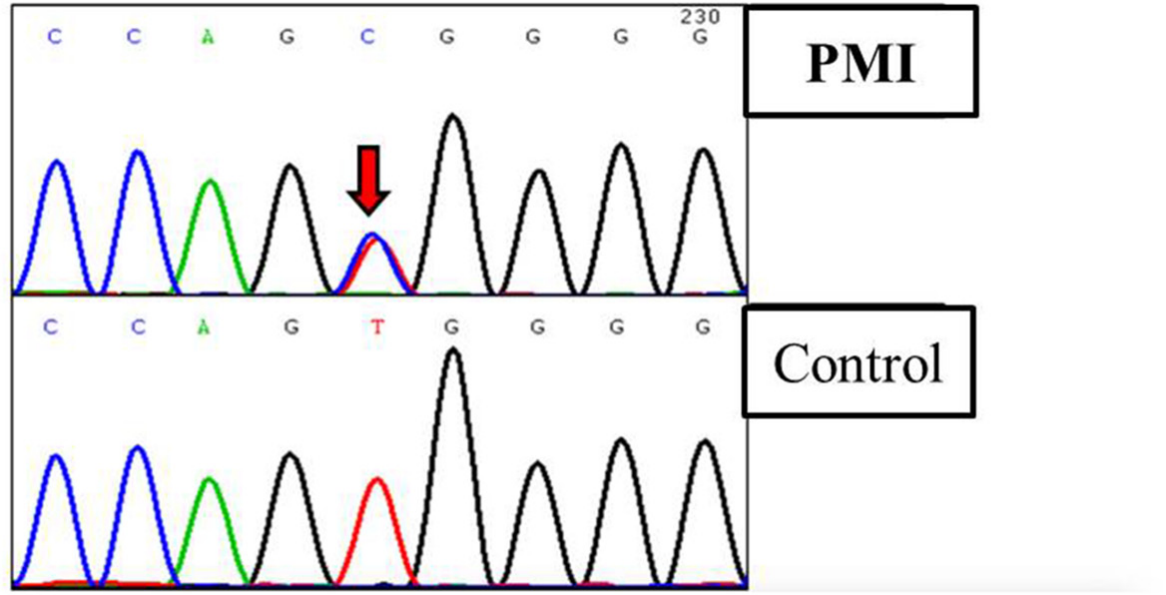
Truncated sequencing chtomatogramof *SCAP* of a patient diagnosed with PMI

**Figure 5:**
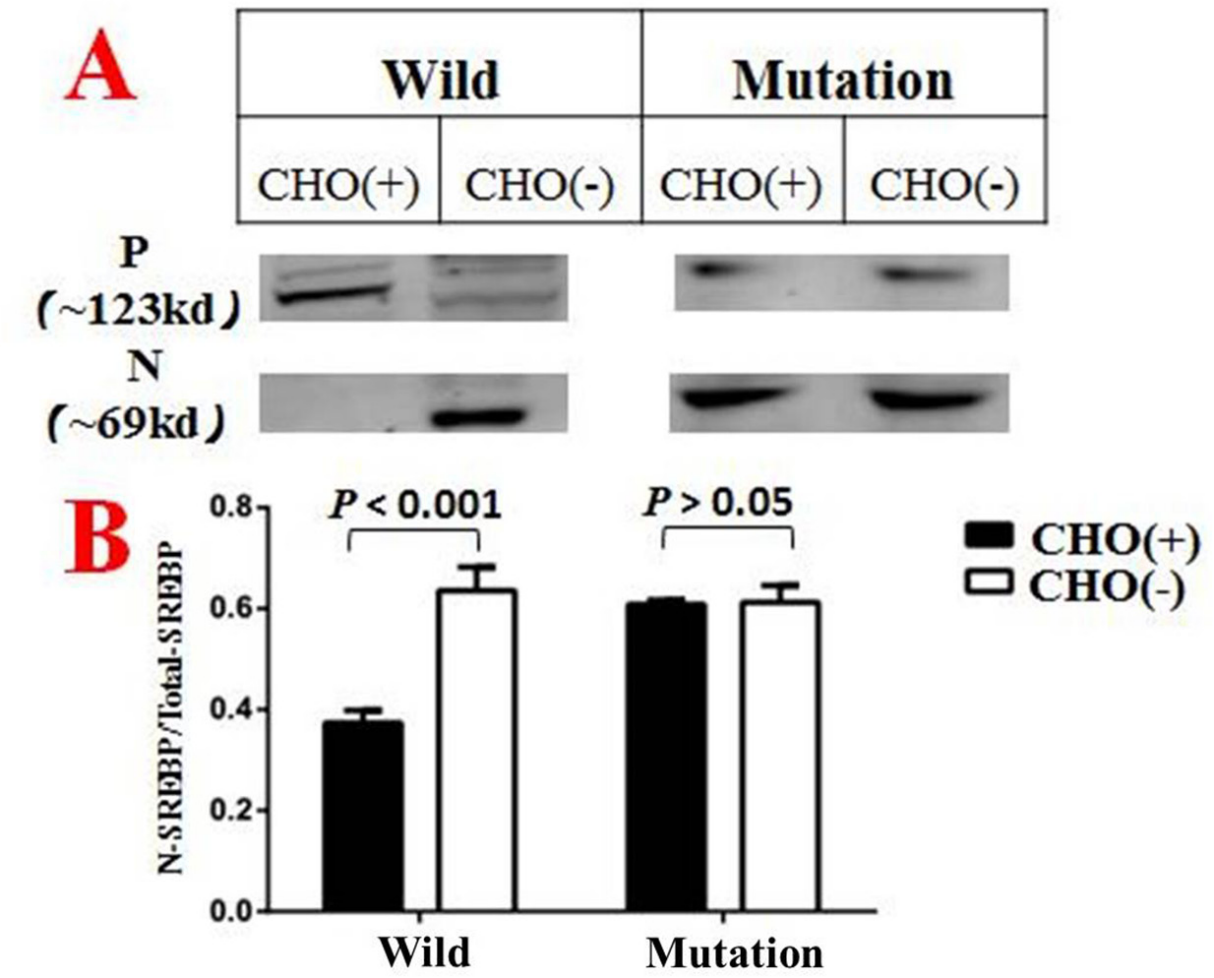
CHO(+) indicated groups treated with medium A1, while CHO(-) indicated groups treated with medium A2 N denotes the N terminal of SREBP that located in the nucleus. P denotes the precursor form of SREBP which located in cytoplasm. In addition, the vertical coordinate in Figure 5B is a ratio calculated by N/N+P. Each column diagram in Figure 5B were calculated from the mean value of three independent biological repeats.

**Figure 6:**
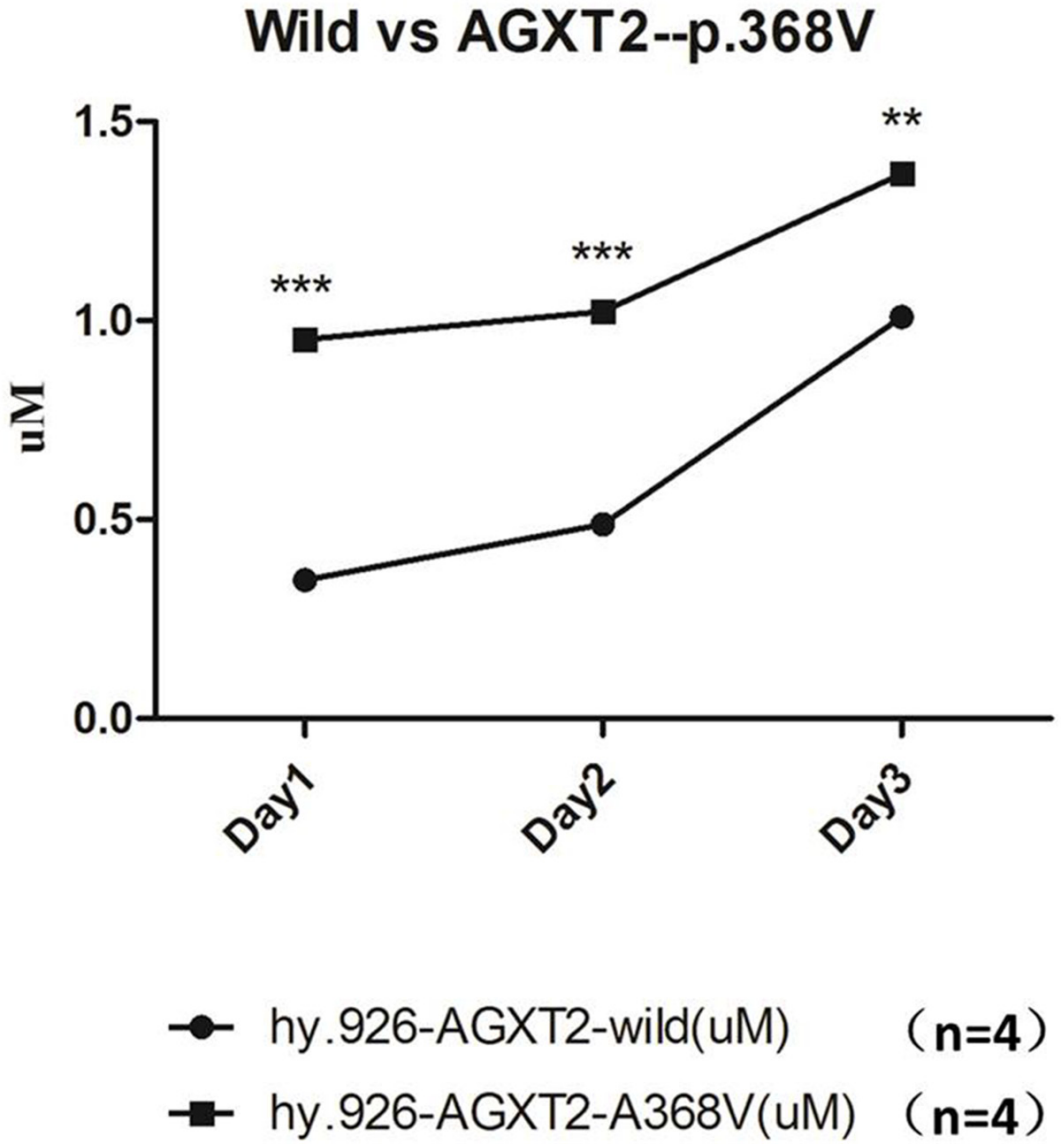
The ADMA level in the conditioned medium of wild type EA. hy926 were significantly higher, compared to that of EA.hy926 cells with mutated *AGXT2* (p.Ala338Val) ^***^ denotes p<0.001; ^**^ denotes p<0.01. Each data point in the line chart were calculated from the mean value of four independent biological repeats.

### Gene screening provided more evidence for disease-related role for *SCAP* but not *AGXT2*

All the 23 exons of *SCAP* and 14 exons of *AGXT2* were screened by sanger sequencing. We found another *SCAP* variants (p.Val468Ala, c. 1403 T>C; Figure 3) on one of the 77 scattered PMI cases, which were predicted to be disease-related based on the facts that (1) the variant was absent in 28, 000 east-Asia population (data abtained from dbSNP of NCBI); (2) the amino acid Val468 of *SCAP* was highly conserved in multiple species. However, we did not found any potential disease-related variant on *AGXT2* of all 77 cases.

### *SCAP*-c.3035C>T (p.Ala1012Val) variant impaired the negative feedback mechanism of cholesterol synthesize in H293T cell lines

*SCAP*-c.3035C>T (p.Ala1012Val) variant was introduced into H293T cell lines by CRISPR-Cas9 methodology. After incubated with medium A (as described in the materials and methods section) for 6 hours, the wild-type goups showed a significant different distribution of SREBP-2 in cytoplasm and nucleus, (Figure 4A) while the *SCAP*-mutated groups shows no such difference (Figure 4B). These phenomenon indicate that the mutated *SCAP*-c.3035C>T (p.Ala1012Val) protein failed to sensing the intracellular cholesterol level, implying a loss of negative feedback mechanism of the mutated *SCAP* coding protein.

### *AGXT2*-c.1103C>T (p.Ala338Val) variant impaired the catabolism of ADMA in EA. hy926 cell lines

*AGXT2*-c.1103C>T (p.Ala338Val) variant was introduced into EA. hy926 cell lines by CRISPR-Cas9 methodology. The conditioned medium was collected at the time point of 24h, 48h and 72h respectively after plant. Elisa analysis showed that the ADMA levels in group harboring a mutated *AGXT2*-c.1103C were significantly (n=4, *P*<0.05) higher than the wild-type group (Figure 5), indicating that the enzyme activity of the mutated *AGXT2* coding protein was significantly impaired.

## DISCUSSION

In the present study, we established an association between *SCAP*/*AGXT2* mutations with PMI in a digenic pattern. Notably, both carriers of digenic mutations were affected with PMI (Figure 1), while individuals with either mutation presented with “normal” MI (n=1) or no MI (n=5). The present study indicated a digenic inheritance of PMI. This finding further support the notion that PMI, as a complex disease and a severe type of CAD, may stem from harboring more than one causative genes at the same time, namely compound heterozygous mutations. The phenomenon that compound heterozygous mutation would cause a more severe phenotype, has been seen often in monogenic diseases [10]. However, former studies on the CAD risk prediction mainly focused on common SNPs [11, 12], which constitute only a small fraction (∼15%) of the heritability of CAD. So the present study may add some more thoughts on the notion that harboring several rare variants at the same time, but not many more comman SNPs, may help predict a higher CAD risk better. Besides, This digenic pattern in the genetic mechanism of MI has also been reported by Erdmann et, a l [13].

While MI often occurs on the basis of atherosclerosis, the genetic mechanisms underlying the progress of coronary atherosclerosis and occlusion from plaque rupture are evidently diverse [14]. Reilly et, al. has shown that the causative genes of stable coronary heart disease (CHD) and MI in the presence of CHD are varied by a two steps GWAS, indicating that some MI-related genes are likely to predispose the initiation and progression of coronary atherosclerosis while some have a specific role in vulnerable plaque and MI [15]. Our study further supports this assumption. *SCAP* is the coding gene of a vital protein in sensing the intracellular cholesterol level and regulating the cholesterol-related genes like *LDLR* and *HMG-CoA* in a negative feedback pattern [16], -17mainly by transfer the SREBPs[17–19]. Zhang et, al. has demonstrated that *SCAP* mutation would abolish its sensor function, which may result in a constitute-activation-like effect of its coding protein [20]. Consequently, the cholesterol synthesis would persist no matter how high the intracellular level would be. This indicates a jeopardized negative feedback mechanism which is vital in maintaining a normal level of cholesterol in blood. In addition, the study of Zhou et, al.’s has shown that when the SREBPs transportation were maintained, by glycosylation of *SCAP* in the study, THP-1 cells would be more prone to transform into foam cells [21]. From these evidences in all, we could make a reasonable inference that *SCAP* mutation would predispose to the pathogenesis of atherosclerosis. Besides, the fact that *SCAP* is associated with non-alcoholic fatty liver disease (NAFLD) [22, 23], a widely accepted risk factor of coronary heart disease [24–26], also support the above conclusion.

As for *AGXT2*, it’s the key enzyme of Asymmetric dimethylarginine (ADMA) metabolism [27]. On the other hand, ADMA is a structural analogue of L-arginine, the accumulation of which would competitive inhibit Nitric Oxide Synthase (NOS), resulting in a reduced synthesis of NO [28]. While NO is the main protection of vascular endothelium as well as the “tranquilizer” of platelet, conditions including genetic factors affecting NO synthesis would significantly impair the endothelial function as well as the stability of platelet, which would predispose to the vulnerable plaque and thrombogenesis [13]. As a result, it is justifiable that *AGXT2* mutation would predispose to MI because of the increased ADMA levels and a subsequent decreased NO synthesis. Besides, *AGXT2* has also been associated with hypertension, a major risk factors of MI [29]. Last but not least, candidate gene study in the attempt to associate *AGXT2* polymorphism with coronary heart disease also showed a positive result [30], although with a low statistical power and no subsequent functional studies. These in all support the potential causative role of *AGXT2* mutation in CAD, or more specifically MI.

### Limitation

In the present study, *in vivo* experiments which could connect two variants with the phenotype of MI directly are lacked, especially when we want to demonstrated the digenic inherited pattern of the two causative genes. Besides, the amount of scattered PMI cases was too small to draw a statistical concrete association. Nevertheless, seeing from the functional studies ahead, this would not undermine the validity of the present conclusions.

## MATERIALS AND METHODS

### Study subjects

In the present study, MI diagnosed (the diagnosis of MI was referred to the Third Universal Definition of Myocardial Infarction.) before the age of 50 for man and 60 for woman was defined as PMI. Family members of a PMI pedigree (n=16, Figure 1) as well as 77 sporadic PMI cases were enrolled under written consents, approved by the Ethics Committee of Peking University People’s Hospital.

### Sample collection and sanger sequencing

Genomic DNA from the index patient and her parents was extracted from whole blood sample in accordance with standard protocols. All samples underwent polymerase chain reaction (PCR) amplification and direct sequencing (Sanger sequencing). PCR products were purified by vacuum pump Axygen PCR. Direct sequencing was carried out with BigDye Terminator DNA sequencing kit (version 3.1) and 3730XL DNA Analyzer. The sequence of PCR primer pairs was based on reference or redesigned using Primer 3 (*LDLR*, NM_000527.4; *SCAP*, NM_012235.3; *AGXT2*, NM_031900.3).

### Whole-exome sequencing and variants filtering

Briefly, exome sequencing was performed at Beijing Novogene Bioinformatics Technology Co., Ltd (Beijing, China). Agilent SureSelect Human All ExonV5 kit was employed to capture Whole-exome sequence. Sequencing was performed on illumina HiSeq4000 platform using PE150 sequencing strategy.

### Cell culture

HEK293T and E. hy926 cells were grown in DMEM (Invitrogen) supplemented with 10% FBS (Invitrogen), 2 mM L-glutamine, 100 U/ml penicillin and 100 ug/ml streptomycin (Invitrogen) at 37°C, 5% CO2.

For the transfection, cells were seeded at a density of 2×10^6^ cells/10 cm plates and transfected with 10-15 ug DNA using Lipofactamine 2000 (Invitrogen), according to the manufacturer instructions.

### Gene modification via CRISPR-Cas9

Experimental procedures were described previously in detail [31]. Wild-type Cas9 plasmid PX458 was obtained from Addgene (plasmid #48138). sgRNAs were synthesized and cloned into PX458, the sequence of guide RNA for *SCAP* and *AGXT2* was designed on http://tools.genome-engineering.org. Donor for introducing the *AGXT2*-variant was a synthesized asymmetric ultramer [32] (Sangon Biotech). For generating *SCAP* and *AGXT2* mutations in H293T and EA. hy926 respectively, we transiently transfected PX458-sgRNA and donor ultramer/DNA by using Lipofectamine 2000 (Invitrogen). After 48 h post-transfection, GFP-positive cells were sorted and re-plated into 0.1% gelatin coated plates at the density of 10, 000 cells per 10cm plate. After 7 days culture, clones were picked under microscope and screened by genomic PCRs and sequencing. Primers and the ultramer were summarized in Supplementary Table 1. Cassette sequences are available on request.

### SREBP-2 processing and western blot analysis

H293T cells line of *SCAP*-c.3035C>T genotype and wild-type control were plated and cultured in growth medium with 10% FBS for 48h. Cells were then incubated for 6 h at 37°C in DMEM with 1% HLDS (Human lipoprotein-deficient Serum) and 10uM atorvastatin (Sigma), which was supplemented with 0 (medium A1) or 50uM (medium A2) MCD/cholesterol to deplete or overload the cells with cholesterol, respectively. Cells were then harvested at 0°C and cell lysates were subjected to 10% SDS polyacrylamide gels as required. Coomassie brilliant blue (CBB) staining was followed the instruction of the Commassie blue fast staining kit (Beyotime).

### ADMA quantitative analysis by Elisa

EA. hy926 cells line of *AGXT2*-c.1103C>T (p.Ala338Val) genotype (n=4) and wild-type control (n=4) were plated and cultured in 6-well dishes with growth medium added by 10% FBS for 24h. Then 1ml conditioned medium from every well were collected every 24 hours for three times for ADMA analysis. Elisa was carried out according to the instructions from the manufactory (Enzo).

### Statistical analyses

The values are reported as mean±SD in the experimental data. The n in the basic experimental data represents number of samples or repetitions. The independent t test was performed for statistical analysis of the differences in the basic experimental data (western blot and Elisa) between pre and post Cholesterol treatment and *AGXT2*-modification.

## CONCLUSION

To sum up, *SCAP* and *AGXT2* were identified to be potential causative genes for PMI. In addition, the present study provided primary data about the possibility of digenic mutation in the causing of PMI.

## SUPPLEMENTARY MATERIALS TABLE


