# Supplementary material for: Digenic mutations on SCAP and AGXT2 predispose to premature myocardial infarction
Source: Oncotarget. 2017 Oct 24;8(59):100141–9. doi: 10.18632/oncotarget.22045 (PMC5725008; doi:10.18632/oncotarget.22045)
Supplement: Supplementary file 1 [file oncotarget-08-100141-s001.pdf]

Digenic mutations on *SCAP* and *AGXT2* predispose to premature myocardial infarction

SUPPLEMENTARY MATERIALS

Supplementary Table 1: Primers and the ultramer used in CRISPR-Cas9

Primers	Sequence (5'--3')
SCAP-sgRNA2-Top	CACCGTATGATTCGATTGACCTTCT
SCAP-sgRNA2-Bottom	AAACAGAAGGTCAATCGAATCATAC
scap-HOMO-1F	AGAGCTGGGAACGACTTTCAG
scap-HOMO-1R	TCCAGCTCTAGGTCAGATGGT
AGXT2-sgRNA5-T	CACCGcaggcatttcgccaaagatt
AGXT2-sgRNA5-B	AAACaatctttggcgaaatgcctgC
AGXT2- HOMO (Ultramer)	gaatgtgtccctctttccacaccttctattttct ccctctagagattg[T]caaatctttggcgaaat gcctgcagcacttTaaTaccttCggagggaaccc catggcctgtgccattggatctgctgt
